# Validation of the Spanish Short Version of the Perception of Humanized Care Behaviors Scale in Hospitalized Adults in Chile

**DOI:** 10.17533/udea.iee.v44n1e04

**Published:** 2026-03-28

**Authors:** Katya Marie Cuadros-Carlesi, Carlos Felipe Henríquez-Roldán, Paola Andrea Ruiz-Araya, Ximena Ruby Alvarado San Román, María Rosa Oyarce Quiroz, María Angélica Vásquez Osses

**Affiliations:** 1 Nurse, Ph.D. Email: katya.cuadros@uvm.cl. Corresponding author. https://orcid.org/0000-0003-4751-815X Universidad de Viña del Mar Chile katya.cuadros@uvm.cl; 2 Statistician, Ph.D. Email: carlos.henriquez@uvm.cl https://orcid.org/0000-0001-6616-1243 Universidad de Viña del Mar Chile carlos.henriquez@uvm.cl; 3 Nurse, Ph.D. Email: paola.ruiz@uvm.cl http://orcid.org/0000-0001-9485-598X Universidad de Viña del Mar Chile paola.ruiz@uvm.cl; 4 Nurse, Ph.D. Email: xalvarado7@gmail.com https://orcid.org/0000-0002-8644-7991 Universidad de Viña del Mar Chile xalvarado7@gmail.com; 5 Statistician, M.Sc. Email: mroq25@gmail.com https://orcid.org/0000-0001-5191-2964 Universidad de Viña del Mar Chile mroq25@gmail.com; 6 Nurse, Ph.D. Email: mvasquez@uvm.cl https://orcid.org/0000-0002-0635-9853 Universidad de Viña del Mar Chile mvasquez@uvm.cl; 7 Faculty of Life Sciences, Universidad Viña del Mar, Chile. Universidad de Viña del Mar Faculty of Life Sciences Universidad Viña del Mar Chile

**Keywords:** nursing care, humanization of assistance, hospitals, reproducibility of results, perception., atención de enfermería, humanización de la atención, Hospitales, estudio de validación, reproducibilidad de los resultados, percepción., cuidados de enfermagem, humanização da assistência, hospitais, reprodutibilidade dos testes, percepção.

## Abstract

**Objective.:**

To validate a new Spanish version of the Perception of Nursing Humanized Care Behaviors Scale (PCHE) in hospitalized adults in Chile.

**Methods.:**

A descriptive, cross-sectional validation study of the PCHE scale was conducted with the participation of adults admitted to four hospitals in Chile. The research was carried out in three stages: (i) linguistic adaptation and content validation through expert judgment; (ii) assessment of internal consistency and preliminary construct validity of the adapted and revised instrument; and (iii) evaluation of the validity and reliability of a proposed abbreviated version of the scale. A total of 1720 patients were selected through non-probabilistic convenience sampling. Following linguistic adaptation and expert review, a pilot test was conducted with 40 patients; after revisions, the 32-item instrument with four response options was applied to a first sample of 344 patients. Subsequently, the response scale was expanded from four to seven options and administered to a second sample of 202 patients. Cognitive interviews and a preliminary Exploratory Factor Analysis (EFA) were also conducted, resulting in a reduction of the instrument from 32 to 15 items. Finally, in a third sample of 1,134 patients, an EFA and a Confirmatory Factor Analysis (CFA) were performed. Internal consistency was assessed using Cronbach’s alpha.

**Results.:**

The original 32-item instrument with four Likert response options showed limitations in item comprehension, relevance, and factorial structure. After adjustments, a 15-item scale was obtained, from which a single dimension-humanized care-emerged, with a Cronbach’s alpha of 0.93.

**Conclusion.:**

The abbreviated PCHE instrument, reduced from 32 to 15 items, is valid and reliable for use in hospitalized adult patients in Chile.

## Resumen

**Objetivo.:**

Validar una nueva versión de la escala en español “Percepción de comportamientos de cuidado humanizado de enfermería” -PCHE- en adultos hospitalizados en Chile.

**Métodos.:**

Estudio descriptivo de corte transversal de validación de la escala PCHE realizado con la participación de adultos ingresados en cuatro hospitales de Chile. La investigación fue desarrollada en tres etapas. (i) adaptación lingüística y validación de contenido por juicio de expertos; (ii) estudio de consistencia interna y validez de constructo preliminar del instrumento adaptado y corregido; y: (iii) estudio de validez y confiabilidad de una propuesta de instrumento abreviado. Participaron 1720 pacientes que fueron seleccionados a través de un muestreo no probabilístico por conveniencia. Luego de la adaptación lingüística y juicio de expertos, se efectúo una prueba piloto en 40 pacientes; y posterior a los ajustes, se aplicó el instrumento de 32 ítems con 4 opciones de respuesta a una primera muestra de 344 pacientes. Posteriormente, se ampliaron las opciones de respuesta de 4 a 7 y se aplicó nuevamente a una segunda muestra de 202 pacientes. Además, se realizaron entrevistas cognitivas y un Análisis Factorial Exploratorio -AFE- preliminar que, derivaron en un instrumento reducido de 32 a 15 ítems. Finalmente, en una tercera muestra de 1134 pacientes, se efectuó AFE y Análisis Factorial Confirmatorio. La consistencia interna se midió por alfa de Cronbach.

**Resultados.:**

El instrumento original con 32 ítems y 4 opciones Likert mostró falencias en la comprensión, pertinencia y estructura factorial de los ítems. Luego de ajustes, se logró obtener una escala de 15 ítems de la que emergió como única dimensión el cuidado humanizado, con un alfa-Cronbach de 0.93.

**Conclusión.:**

La propuesta del instrumento PCHE abreviado de 32 a 15 ítems es válida y confiable para ser utilizado en pacientes hospitalizados chilenos.

## Resumo

**Objetivo:**

Validar uma nova versão em espanhol da escala Percepción de Comportamientos de Cuidado Humanizado de Enfermería (PCHE) em adultos hospitalizados no Chile.

**Metodos.:**

Estudo descritivo, transversal, de validação da escala PCHE, realizado com a participação de adultos internados em quatro hospitais do Chile. A pesquisa foi conduzida em três etapas: (i) adaptação linguística e validação de conteúdo por meio de julgamento de especialistas; (ii) avaliação da consistência interna e da validade de construto preliminar do instrumento adaptado e revisado; e (iii) estudo de validade e confiabilidade de uma versão abreviada proposta. Participaram 1720 pacientes selecionados por amostragem não probabilística por conveniência. Após a adaptação linguística e o julgamento de especialistas, realizou-se um teste piloto com 40 pacientes; posteriormente, aplicou-se o instrumento de 32 itens com quatro opções de resposta a uma primeira amostra de 344 pacientes. Em seguida, as opções de resposta foram ampliadas de quatro para sete e o instrumento foi novamente aplicado a uma segunda amostra de 202 pacientes. Além disso, foram realizadas entrevistas cognitivas e uma Análise Fatorial Exploratória (AFE) preliminar, o que resultou na redução do instrumento de 32 para 15 itens. Por fim, em uma terceira amostra de 1.134 pacientes, foram conduzidos AFE e Análise Fatorial Confirmatória (AFC). A consistência interna foi avaliada pelo alfa de Cronbach.

**Resultados.:**

O instrumento original, composto por 32 itens com quatro opções de resposta Likert, apresentou limitações quanto à compreensão, pertinência e estrutura fatorial dos itens. Após os ajustes, obteve-se uma escala de 15 itens, da qual emergiu uma única dimensão - o cuidado humanizado - com alfa de Cronbach de 0.93.

**Conclusão.:**

A versão abreviada da escala PCHE, reduzida de 32 para 15 itens, mostrou-se válida e confiável para utilização em pacientes adultos hospitalizados no Chile.

## Introduction

Nursing is centered on people, and humanization of care is one of the fundamental pillars of its work. However, in some clinical contexts, a growing depersonalization on part of the nursing team has been evidenced, resulting in undignified treatment and dehumanized patient care, the latter understood as mechanical and eminently technical care, accompanied by discriminatory attitudes and the inappropriate use of verbal language.^1^ Consequently, care is focused on the organic dimension of the disease, limiting contact with the patient exclusively to the execution of procedures, in detriment of a comprehensive and person-centered approach.^2^ Faced with this progressive loss of the human dimension in care, the concept of "humanization" has emerged strongly, understood as an ethical and professional response to the process of dehumanization. In the field of nursing, humanization implies the conscious and reflexive use of the professional's own humanity to care for and accompany the experience of others.^3^

From this perspective, Paterson and Zderad^4^ conceive human caring as an existential experience and a "reciprocal call and response", in which patient and nurse meet as human beings. The patient calls the nurse with the expectation of being cared for, and the nurse responds by virtue of her need to care for another. Watson defines human caring as a practice based on loving kindness and equanimity, requiring authentic presence, a deep belief in others and the cultivation of personal spirituality as a pathway to wholeness of body, mind and spirit. This type of care is oriented towards respecting individual identity and values, promoting uniqueness, autonomy and the development of human potential.^5^ In this context, assessing patients' perceptions of humanized care becomes fundamental, as it allows us to identify strengths and weaknesses in care practice, improve the quality of care, and strengthen the therapeutic bond between the professional and the patient.^6^ To this end, various instruments have been developed to empirically measure this concept through observable indicators of humanization,^7^ one of the most widely used being the "Perception of Humanized Nursing Care Behaviors" (PCHE by its acronym in Spanish ) questionnaire, developed for the first time in Colombia,^8^ whose theoretical conception of humanization was based on Jean Watson's theory of human care.

In Chile, cultural adaptations and validation studies of the PCHE have been carried out. Currently, the fourth version of the instrument is available, which restricts its application to self-administration. However, this process was carried out on small samples of patients, using traditional construct validation techniques based on Pearson's correlation_._^9^ Furthermore, the past-tense wording of the items restricts their application to hospital discharge or post-discharge, which prevents their use during hospitalization, a time when the perception of care may be more immediate, authentic and less affected by selective recall. It should be noted that, at discharge, patients are often exposed to multiple cognitive and emotional demands, such as understanding medical indications, organizing the therapeutic and dietary regimen, coordinating subsequent controls, as well as waiting for relatives, situations that could affect both the willingness to answer a questionnaire and the quality and depth of their responses.

On the other hand, the self-administration modality does not allow for the application of the instrument to illiterate people, with low schooling or with physical limitations that could be overcome with the support of another person from outside the research team to act as a witness of faith, if required. Faced with this scenario, it is necessary to have a new version of the questionnaire that allows its application in hospitalized patients, with items written in the present tense, applicable in a self-administered or hetero-administered manner. This motivates this study which aims to validate a new version of the questionnaire "Perception of humanized nursing care behaviors" for Chile.

## Methodology

A descriptive cross-sectional methodological study carried out in three stages. Stage 1 considered the linguistic adaptation, grammatical correction of the instrument, content validation by expert judgement and pilot testing. Stage 2 corresponded to the study of internal consistency and preliminary construct validity of the adapted and corrected instrument. In Stage 3, the final validity and reliability study of a new instrument proposal was carried out. The research was carried out in four hospitals in Chile; three located in the Valparaíso region and one in the Metropolitan region. Data collection took place between November 2023 and May 2025. Three of the facilities are of high complexity and one of low complexity. Considering the three stages of the study, a total of 1,720 adult patients hospitalized in medical-surgical units participated through non-probabilistic convenience sampling. In the first stage, a pilot test was conducted with 40 hospitalized patients. For the second stage, 546 patients were included, taking into consideration Campo-Arias and Oviedo,^10^ who indicate that between 5 and 20 subjects are required for each item of an instrument. In the third stage, we worked with a sample of 1134 patients. This decision was based on the recommendations of Comrey and Lee,^11^ who consider that sample size greater than 1,000 cases allows for "excellent" results in confirmatory factor analysis (CFA). Furthermore, given that CFA was conducted through polychoric correlations between items measured on a 7-point Likert scale, it was necessary to apply structural equation modelling (SEM) to obtain goodness-of-fit measures. In this context, Kline^12^ *indicates that a minimum sample size of 1000 participants are required* to meet the assumptions and to obtain stable estimates in models with ordinal latent variables. Consequently, the research team defined the objective of gathering at least 1,100 participants for this phase of the study, with the purpose of guaranteeing a robust validation of the instrument, considering that there were already four previous versions validated in Chile. This decision sought strengthen the reliability and validity of the tool, ensuring its relevance and applicability to the national community. Inclusion criteria were established as follows: patients aged 18 years or older who were hospitalized in non-critical units, without alterations in consciousness or cognition; in the absence of discomfort (pain, invasive procedures or others) that would hinder their participation in the study; the presence of environmental conditions that ensured the privacy of the responses; and voluntary acceptance of participation, formalized by signing the informed consent form.

The Perception of Humanized Nursing Care Behaviors (PCHE) questionnaire was used, the fourth version of which was cross-culturally validated for Chile. It consists of three dimensions grouping 32 items: qualities of nursing (7 items), openness to nurse-patient communication (8 items) and willingness to care (17 items). The global perception of humanized care is evaluated based on a measure of perception in four categories of scores between 32 and 128 points, obtained by adding the Likert scale scores of all the items of the questionnaire, with four response alternatives: 1= never, 2= sometimes, 3= almost always and 4= always.^9^

The details of the three aforementioned stages are described below: 

*Stage 1*

As a first step, the questionnaire was evaluated by two linguistic experts who made grammatical adjustments to the items and wrote the questions in the present indicative tense, so that the questionnaire could be applied both at the time of hospitalization and at discharge prior to leaving the hospital. Then, a validation process was carried out by five experts, and the content validity index was calculated.^13^ Subsequently, a pilot test was carried out on 40 patients hospitalized in medical-surgical services; to check their understanding of the questionnaire, response times and other aspects associated with the conditions of application. 

*Stage 2*

For the study of internal consistency and construct validity, the questionnaire was applied to two samples of patients. The first sample consisted of 344 patients, and the second of 202, respectively. These samples were not randomly selected, but patients who were hospitalized and met the inclusion criteria were invited to participate. For data collection, the researchers entered the rooms, identified themselves as researchers belonging to the Universidad Viña del Mar and carried out an assessment of the patients' general and cognitive state. They then explained the objectives of the study and invited them to participate, asking them to sign an informed consent form. To assess how respondents understood, interpreted and responded to the questionnaire items, 73 cognitive interviews were conducted. This qualitative technique allows us to explore how respondents understand, interpret and respond to the items in a questionnaire, which allows us to identify formulation problems such as ambiguity, inadequate vocabulary or confusing grammatical structures that affect the validity of the responses.^14^ These interviews allowed us to explore how participants interpreted each questionnaire item, with emphasis on the processes of comprehension, retrieval, judgement and response formulation, in accordance with the cognitive processing model of Tourangeau, Rips and Rasinski.^15^ The interview technique used was the verbal survey.^16^ As part of the cognitive interview, the patients were asked to indicate which of two or more similar items they understood best, so that they could be included in the final instrument of the next stage. Given the observed incongruence between the initial content validity (expert judgment) and the evidence of cognitive misunderstanding by the patients, the decision was made to prioritize construct validity and practical applicability. Consequently, a new expert judgment was not sought, recognizing that the instrument's functionality and the adequate measurement of the construct in the real-world context are paramount for the final validity of the instrument.^17^

The questionnaire was administered in an assisted or self-administered manner, according to the patient's preference and in the absence of health personnel inside the room, taking care to ensure the privacy of their responses. When the questionnaire was administered in an assisted manner, patients were shown the printed response options in a table in ruler format was implemented with the new Likert scale with its numbering, and equivalence in words: 1= "never"; 2= "rarely"; 3= "occasionally"; 4= "sometimes"; 5= "frequently"; 6= "very often"; and 7= "always", so that they could indicate their preference on the printed sheet, thus guaranteeing the privacy of their responses, especially when they were hospitalized in rooms shared with other patients. In addition, the choice of seven response alternatives gave patients the possibility of having a wider range of options for each of the items consulted.

Data collection was carried out during hospitalization or at discharge, recording both the day of hospitalization on which the questionnaire was administered and the status of "hospitalized" or "discharged".

Once collected, the questionnaires were coded, and the data were transferred to an Excel spreadsheet to be exported and processed in Stata version 18. A descriptive analysis of the data consisted in calculating frequencies, and position and dispersion indicators for each item. The internal consistency of the questionnaire was assessed using Cronbach's Alpha coefficient. To assess the construct validity of the instrument, an exploratory factor analysis (EFA) was carried out using polychoric correlations.^17^ This is the appropriate statistical technique for quantifying the association of variables measured on a Likert scale. It is used to explore and understand the underlying structure of a set of variables, particularly when these are ordinal or categorical. This strategy was used because classical factor analysis techniques based on Pearson's coefficient are based on continuous variables with a k-variate normal distribution (k represents the number of items). Moreover, probabilistic behavior must be symmetric with respect to their means. Polychoric correlations do not require the variables to be continuous, to follow a normal distribution and do not require a symmetrical distribution. Likert scales are ordinal categorical variables. Kiwanuka et al.^18^ points out that with asymmetric responses with few alternatives the results will not be stable and that five or more response options will improve the estimates.

The use of polychoric correlations allows factor analysis to provide a more accurate representation of the underlying relationships between ordinal variables, which improves the estimation of latent factors and ensures that relationships are not distorted by the lack of continuity of variables.^19^ Polychoric correlations between items were calculated and factors whose eigenvalues were greater than one were retained. According to suggested by some authors, an item was assigned to a sum-factor scale if its factor loading was greater than 0.50 and its differences with other factor loadings of this item were greater than 0.20.^20^ Confirmatory factor analysis (CFA) was then performed and implemented in the same software. Goodness-of-fit measures are not directly implemented when conducting CFA with polychoric correlations; therefore, the measures suggested by Finch & French^21^ were followed and calculated through structural equations: root mean square error of approximation (SRMR), residual root mean square error of approximation (RMSEA), Tucker-Lewis (TLI) and comparative fit index (CFI). Values lower than 0.05 for the SRMR and RMSEA indicate a good model fit and values higher than 0.90 for the TLI and CFI indicate that the model is adequate.

The study was approved by the Scientific Ethics Committees of the Aconcagua Health Service, the Military Hospital of Santiago and the University of Viña del Mar. Likewise, Dr. Angélica Melita authorized the use of the fourth version of the PCHE validated for Chile. All patients signed an informed consent form to participate in the study.

## Results

Overall, Table 1 indicates that the patient sample was quite homogeneous with respect to age, days of hospitalization, and educational level, except for the initial stage, which included a limited number of participants. Most patients (56%) were hospitalized in Surgical Units, while 38% were hospitalized in Medical Units. It is worth noting that 18% of patients had a low educational level (illiterate or incomplete basic education). In addition, 11% had one day of hospitalization at the time the questionnaire was applied.

**Table 1:** Characterization of the patients participating in the study by research stage

	Stage			
Variables	1 *n*=40	2 *n*=546	3 *n*=1134	Total *n*=1720
**Participants per unit; *n* (%)**				
Medical	5 (12.5)	197 (36.1)	446 (39.3)	648 (37.7)
Surgical	33 (82.5)	302 (55.3)	622 (54.9)	957 (55.6)
Medical- Surgical	2 (5.0)	47 (8.6)	66 (5.8)	115 (6.7)
**Sexo; *n* (%)**				
Masculino	20 (50.0)	283 (51.8)	622 (54.3)	925(53.4)
Femenino	20 (50.0)	263 (48.2)	524 (45.7)	807(46.6)
Age (years)				
Mínimum	20	18	18	18
Median	61	60	64	63
Average	58	57	61	60
Standard deviation	19	18	17	17
Máximum	88	93	99	99
No information	0	2	1	3
**Level of education; *n* (%)**				
Illiterate	2 (5.0)	18 (3.3)	12 (1.0)	32 (1.9)
Incomplete basic education	9 (22.5)	97 (17.8)	172 (15.2)	278 (16.2)
Basic completed	6 (15.0)	93 (17.0)	147 (13.0)	246 (14.3)
Secondary incomplete	8 (20.0)	78 (14.3)	134 (11.8)	220 (12.8)
Secondary completed	8 (20.0)	152 (27.8)	329 (29.0)	489 (28.4)
Higher education student	6 (15.0)	70 (12.8)	213 (18.8)	289 (16.8)
Completed higher education	1 (2.5)	35 (6.4)	126 (11.1)	162 (9.4)
No information	0 (0.0)	3 (0.6)	1 (0.1)	4 (0.2)
				
Days of hospitalization				
Mínimun	1	1	1	1
Median	4	5	5	5
Average	9	9	9	9
Standard deviation	19	16	15	16
Máximum	120	210	210	210
No information	0	3	34	37

The breakdown of the results by stage is as follows:

Stage 1. Linguistic adaptation, grammatical correction of the instrument and content validation by expert judgement

The nursing experts agreed on the suitability of the instrument for assessing the humanized care behavior of nursing staff. A content validity index of 0.97 was obtained. Regarding face validity, the experts considered the instrument to be clear and relevant. The pilot study, carried out on a sample of 40 patients, revealed limitations in the understanding of some items, as well as difficulties associated with its application. Among the factors that hindered the self-application of the instrument, the following were identified in the patient: the presence of medical devices, the decubitus position during rest and the absence of corrective lenses. Although patients expressed their willingness to participate in the study, when informed about the self-application modality, a small number of patients wanted to participate by answering the instrument themselves; however, the vast majority asked for help in recording their answers. Consequently, it was decided to implement an assisted mode of application, in which the researcher read the items aloud and recorded the responses provided by the patient.

Stage 2. Study of internal consistency and preliminary construct validity of the adapted and corrected instrument

The questionnaire was administered to 546 patients across two complementary samples of 344 and 202 patients, respectively. In the initial sample of 344 patients, difficulties were observed regarding both the semantic comprehension of certain items and the participants' availability of information needed to respond adequately. These difficulties made it necessary to provide additional explanations to patients about the content of the items, which involved exemplifying the statements in the specific context of nursing care during hospitalization, with the aim of facilitating their understanding and interpretation. In addition, the four- alternative Likert scale made data capture more complex, as most patients concentrated their responses on two options: "almost always" and "always". The latter affected the statistical analysis of the data - especially in the calculation of correlations - which affected the study of construct validity, given the low variability of the results. This ruled out the calculation of traditional correlations (Pearson, Spearman and Kendall). Despite not complying with the minimum principle of five response alternatives, EFA was explored using principal component analysis through polychoric correlations. This allowed a preliminary visualization of three dimensions that explained 75.4% of the variability in the data (Table 2). However, these three dimensions did not group the items as reported for the fourth Chilean version of the questionnaire; since, 29 items were grouped under the first factor, two (items 22 and 30) under the second factor: "they respond in a timely manner to your call" and "they tell you that when you need something, you can call them". Finally, item 3 was the only item that was grouped in the third factor: "they show interest in providing you with comfort during your hospitalization". When assessing internal consistency, Cronbach's Alpha = 0.9643 was obtained.

**Table 2:** Eigenvalues and variance explained by applying EFA on the matrix with polychoric correlations with the original instrument of 32 items and 4 response alternatives on a Likert scale using the first sample (*n* = 344)

Factor	Eigen-value	Proportion of Explained Variance	
		Individual	Cumulative
1	21.88	0.68	0.68
2	1.27	0.04	0.72
3	0.99	0.03	0.75
4	0.76	0.02	0.78
5	0.73	0.02	0.80
6	0.69	0.02	0.82
7	0.64	0.02	0.84
8	0.64	0.02	0.86
9	0.51	0.02	0.88
10	0.47	0.01	0.89
11	0.41	0.01	0.91
12	0.39	0.01	0.92
13	0.32	0.01	0.93
14	0.30	0.01	0.94
15	0.28	0.01	0.95
16	0.27	0.01	0.95
17	0.25	0.01	0.96
18	0.25	0.01	0.97
19	0.21	0.01	0.98
20	0.17	0.01	0.98
21	0.13	0.00	0.99
22	0.13	0.00	0.99
23	0.10	0.00	1.00
24	0.08	0.00	1.00
25	0.08	0.00	1.00
26	0.07	0.00	1.00
27	0.05	0.00	1.00
28	0.03	0.00	1.00
29	0.03	0.00	1.00
30	-0.01	0.00	1.00
31	-0.02	0.00	1.00
32	-0.06	0.00	1.00

Based on the above, it was decided to extend the Likert scale to seven alternatives with a scale like that used by the Chilean school grading system and a preliminary exploratory study was carried out with a second sample of 202 patients to observe the behavior of the modifications. PCA was performed through principal component analysis using polychoric correlations, which revealed a unidimensional structure (Table 3). The first eigenvalues -above 1: 21.35 and 1.30- explained 70.8 % of the variability in the data. The third eigenvalue was 0.91 and contributed 2.9% to the variability explained. The internal consistency measured by Cronbach's alpha was 0.97.

**Table 3:** Polychoric correlations of the 32 items of the original instrument with 7 response alternatives on a Likert scale with the first 3 factors. Exploratory study with the second sample of 202 patients

Item	Factor		
	1	2	3
1-They make you feel like a person.	0.67	-0.33	0.23
2-You are treated with kindness.	0.71	-0.43	0.27
3-They show interest in providing you with comfort during your hospitalization.	0.76	0.15	0.24
4-They look you in the eyes when they talk to you.	0.73	0.29	0.30
5-They spend time with you to clarify your concerns.	0.81	0.34	0.27
6-They make you feel well cared for when they talk to you.	0.77	0.04	0.32
7-They make you feel at ease when they are with you.	0.72	-0.16	0.36
8-You feel confident when you are cared for.	0.76	-0.12	-0.04
9-They give you the space to talk to them.	0.75	0.34	0.08
10-They explain in advance the procedures they will carry out.	0.71	0.24	-0.01
11-They respond confidently and clearly to your questions.	0.76	0.24	0.07
12-They identify themselves by name and position before performing procedures on you.	0.55	0.60	0.11
13-They devote the time required for your care.	0.76	0.23	0.16
14-You are given explanations about your health care when you ask for them.	0.70	0.44	0.03
15-They explain care to you in a calm tone of voice.	0.79	0.16	-0.07
16-They call you by your name	0.63	-0.48	0.15
17-They show you respect for your beliefs and values.	0.68	-0.13	0.11
18-They attend to their basic needs (hygiene, urinary and bowel movements) in a timely manner	0.75	-0.13	-0.01
19-You are provided with sufficient and timely information so that you can make decisions about your health situation.	0.74	0.45	0.28
20-They tell you that they are attentive to your needs.	0.81	0.33	0.06
21-They allow you to express your feelings about illness and treatment.	0.70	0.36	0.12
22-They respond in a timely manner to your call.	0.77	0.09	-0.16
23-They identify their physical, psychological and spiritual needs.	0.76	0.29	-0.19
24-They listen to you attentively.	0.84	0.12	-0.04
25-They ask and worry about your state of mind.	0.74	0.39	0.08
26-They provide you with warm and gentle care.	0.82	0.20	-0.06
27-They help you manage your physical pain.	0.70	0.08	-0.38
28-They show you that they are responsible for your care.	0.81	-0.05	-0.14
29-They respect your decisions.	0.75	0.01	0.01
30-They tell you that when you need something, you can call them.	0.80	-0.05	-0.12
31-They respect your physical privacy.	0.76	0.01	-0.18
32-You are given the medicines prescribed by the doctor on time.	0.69	-0.05	-0.58

This revealed that all 32 items were highly correlated with the first factor, suggesting that the items concentrated primarily on a single dimension. This finding, compounded by observations from the cognitive interviews with patients-namely, difficulties in comprehension, ambiguity, item similarity, and the lack of information to provide an answer-led to the elimination of 17 items based on four distinct arguments, as detailed below. The original item number from the fourth 32-item version is indicated in brackets, "[Item #]"

1. **Lack of semantic clarity (difficulty in understanding the wording or vocabulary).** Items whose language was not understood by participants due to ambiguity, technicality, or unfamiliarity with certain terms were removed. For example, "[Item 17] They show you respect for your beliefs and values" generated confusion due to the vagueness of the concepts involved, which were interpreted in different ways (such as religion, personal customs, among others). Likewise, "[Item 15] They explain care in a calm tone of voice" presented difficulties because patients did not clearly understand what "calm" meant. A similar situation occurred with "[Item 26] They give you warm and gentle care", where the words "warm" and "gentle" were considered subjective and imprecise. Finally, "[Item 19] They provide you with sufficient and timely information to enable you to make decisions about your health situation" was considered long and confusing, and the content was not clearly associated with the role of nurses.

2. **Lack of judgement (patients are unable to assess behavior).** Items whose assessment required technical, clinical or administrative knowledge that patients did not possess were eliminated, making a valid response difficult. For example, "[Item 32] You are given the medicines prescribed by the doctor on time" was considered difficult to answer because patients did not know the administration schedules. In the case of "[Item 23] Identify your physical, psychological and spiritual needs", patients indicated that they did not have access to the clinical reasoning of the staff, especially regarding psychological or spiritual dimensions. The same was true for "[Item 13] They dedicate the required time to their care", as respondents did not know what the "adequate" time was to assess whether what they received was sufficient. In these cases, the formulation of a judgement became subjective or speculative, which compromised content validity.

3. **Redundancy or semantic overlap between items**. Pairs or trios of items were identified that addressed similar content, generating confusion among respondents. In response to this, redundant items were eliminated and the item that showed the greatest clarity and discriminatory ability was retained. For example, "[Item 30] They tell you that when you need something, you can call them" and "[Item 20] They tell you that they are attentive to your needs" were eliminated because they were similar and abstract; instead, "[Item 22] They respond to your call in a timely manner" was kept because it was more concrete and evaluable. Another case was "[Item 6] They make you feel well cared for when they talk to you", "[Item 7] They make you feel calm when they are with you", and "[Item 8] They make you feel confident when they take care of you". Patients indicated that all three were very similar, and the last one was retained as it was more comprehensive and understandable. Finally, "[Item 9] They give you space to talk to them" and "[Item 5] They take time to clarify your concerns" were also removed, as patients indicated that they overlapped with "[Item 24] They listen to you attentively", which was finally retained for its greater clarity.

4. **Disconnection with the reality of care (lack of contextual validity).** Some items were removed for not adequately reflecting patients' actual experience in the hospital setting or for attributing behaviors to nurses that were not recognized as part of their role. “[Item 12] They identify themselves by name and position before performing procedures" was perceived as unrealistic, as patients indicated that this presentation occurred only at the beginning of care and not before each procedure. Therefore, its omission was not understood as a lack of humanization of care. As for "[Item 29] Your decisions are respected", there was confusion as to the type of decisions referred to, as patients indicated that in hospitalization they were not always asked to decide, or they associated this responsibility with the doctor. Likewise, "[Item 19] They provide you with sufficient and timely information so that you can make decisions about your health situation" was perceived as being disconnected from the role patients assign to the nursing team. Finally, "[Item 3] They show interest in making you comfortable during your hospitalization" and "[Item 28] They show you that they are responsible for your care" were eliminated because the concepts of "interest" and "responsibility" were considered abstract and difficult to exemplify from concrete experiences.

Stage 3: final validity and reliability study of a new instrument proposal

Considering that the items had previously been validated by experts without reservations and recognizing that this validation demonstrated significant discrepancies when compared with the surveyed patients, the decision was made to proceed directly to construct validation. In this stage, the questionnaire was applied to 1134 patients. Of these, 8% (*n* = 86) were interviewed at discharge and 92% (*n* = 1048) during hospitalization. The patient's preferred mode of application was assisted (97%, *n* = 1095). Only 3% (*n* = 32) responded autonomously. The newly proposed instrument with 15 items and seven response options was subjected to a construct validity study. The EFA applied on the polychoric correlations retained only one factor that explained 66.5% of the variability (Table 4).

**Table 4:** Eigenvalues and variance explained by applying Exploratory Factor Analysis on the matrix with polychoric correlations with the modified instrument with 15 items and 7 Likert- scale response options (*n* = 1134)

Factor	Eigen-value	Proportion of Explained Variance	
		Individual	Cumulative
1	9,98	0.67	0.67
2	0,79	0.05	0.72
3	0,56	0.04	0.76
4	0,50	0.03	0.79
5	0,47	0.03	0.82
6	0,42	0.03	0.85
7	0,37	0.03	0.87
8	0,33	0.02	0.89
9	0,31	0.02	0.91
10	0,26	0.02	0.93
11	0,25	0.02	0.95
12	0,29	0.02	0.96
13	0,20	0.01	0.98
14	0,19	0.01	0.99
15	0,16	0.01	1.00

The overall internal consistency of this proposed new instrument with 15 items was Cronbach's Alpha = 0.93 (Table 5).

The EFA indicated that item 8, which has traditionally been linked to humane treatment of patients, had a factor load of less than 0.5, so internal consistency and CFA calculations were performed with and without its inclusion in the instrument, showing that its retention did not negatively affect the indicators (Tables 5 and 6). The final questions of the validated Spanish instrument PCHE (Percepción de Comportamientos de Cuidado Humanizado) can be found in the appendix of this article.

**Table 5:** Consistency of the modified instrument with 15 items and 7 response alternatives on a Likert scale. *n* = 1134

Item	14 items (excluding item 8)					15 items		
	Item-test	Item-rest	Interitem			Item-test	Item-rest	Interitem	
	Corr.	Corr.	Cov.	Alpha		Corr.	Corr.	Cov.	Alpha
1-They make you feel like a person.	0.68	0.64	0.72	0.93		0.68	0.64	0.65	0.93
2-You are treated with kindness.	0.72	0.68	0.72	0.93		0.72	0.69	0.65	0.93
3-They look you in the eyes when they talk to you.	0.72	0.67	0.70	0.93		0.73	0.68	0.63	0.93
4-You feel confident when you are cared for.	0.74	0.70	0.71	0.93		0.74	0.70	0.64	0.93
5-They explain in advance the procedures they will carry out.	0.75	0.70	0.69	0.93		0.74	0.69	0.62	0.93
6-They respond confidently and clearly to your questions.	0.79	0.74	0.68	0.93		0.78	0.74	0.62	0.93
7-You are given explanations about your health care when you ask for them.	0.76	0.71	0.67	0.93		0.76	0.71	0.61	0.93
8-They call you by your name	-	-	-	-		0.42	0.37	0.69	0.93
9-They attend to their basic needs (hygiene, urinary and bowel movements) in a timely manner.	0.71	0.66	0.70	0.93		0.71	0.66	0.63	0.93
10-They allow you to express your feelings about illness and treatment.	0.75	0.69	0.66	0.93		0.75	0.69	0.60	0.93
11-They respond in a timely manner to your call.	0.75	0.70	0.68	0.93		0.75	0.70	0.61	0.93
12-They listen to you attentively.	0.83	0.79	0.68	0.93		0.83	0.80	0.61	0.92
13-They ask and worry about your state of mind.	0.76	0.70	0.66	0.93		0.76	0.70	0.60	0.93
14-They help you manage your physical pain.	0.73	0.68	0.69	0.93		0.72	0.68	0.63	0.93
15-They respect your physical privacy.	0.67	0.62	0.71	0.93		0.67	0.62	0.64	0.93
Test scale			0.69	0.934	Mean (unstandarised items)			0.63	0.932

Indirect descriptive measures of goodness-of-fit with the theoretical model (CFA) were obtained through structural equations and are presented in Table 6. The appropriate model was obtained with a dimension consisting of 15 items with seven alternatives.

**Table 6:** Fit indicators of the modified PCHE instrument, 15-item instrument with 14 items (excluding item 8: "They call you by your name") n=1134

Fit indicator		Criterion	Instrument	
			15 items	14 items
Likelihood ratio				
chi2_ms(77)	model vs saturated	Significant *p*-values expected	842.122	772.827
p > chi2			0.000	0.000
chi2_bs(91)	basal vs saturated		9481.347	9235.207
p > chi2			0.000	0.000
Population error				
RMSEA	Root Mean Squared Error of Approximation	< 0.07	0.086	0.089
90% CI lower bound			0.081	0.084
upper bound			0.091	0.095
Approximate *p*	Probabilidad RMSEA ≤ 0,05		0.000	0.000
SRMR	Standardised Root Mean Square Residual	< 0.08	0.042	0.041
Information criterion				
AIC	Akaike's Information Criterion		43126.28	40921.45
BIC	Bayesian Information Criterion		43352.78	41132.85
Baseline comparaisons				
CFI	Comparative Fit Index	> 0.90	0.920	0.924
TLI	Tucker-Lewis Index	> 0.90	0.906	0.910

## Discussion

Although initially linguistic and grammatical adjustments were made to the fourth Chilean version of the questionnaire "Perception of Humanized Care Behaviors of Nursing" (PCHE) -for the purpose of being applied to hospitalized patients with a minimum stay of 24 hours- empirical background information emerged that motivated the research team to rethink structural aspects of the instrument. Limitations related to semantic clarity of some items, difficulties for patients to make valid judgements, redundancy in content and lack of contextual appropriateness were identified. In addition, it was recognized that the original format, consisting of 32 items and a four-choice Likert scale, was not conducive to the quality and efficiency of the data collection process. Based on this, it was decided, firstly, to modify the response scale to a seven-point Likert scale, and secondly, to reduce the total number of items to 15. Several studies have documented that shorter instruments tend to increase the response rate^22^ and improve the validity of responses, as they reduce respondent fatigue and encourage more conscious reflection.^23^It should be noted that the fourth version of the instrument was designed to be administered at the time of discharge from hospital;^9^ this considerably limited the number of eligible patients per day, making it difficult to obtain representative samples and requiring considerable man-hours to administer. The modified version was administered both during hospitalization and at the time of discharge, which allowed for broader participation coverage. This strategy not only facilitated the expression of perceptions in real time, but also reduced recall bias, providing more reliable data for continuous improvement of quality of care.

On the other hand, given the current trend towards short-term hospitalizations, in some cases less than 24 hours,^24^ it is necessary to review the exclusion criteria for patients with less than a full day's stay. In this study, 195 patients (11.3%) with at least 24 hours of hospitalization were included and were not unable to make valid judgements about the care received. This suggests that future research could explore the applicability of the instrument in this subgroup. On the other hand, by using a hetero-assisted application format, we ensured the inclusion of patients with low educational attainment or functional illiteracy, who are usually excluded from studies that require reading skills. As has been described in the literature,^25^ interviewer- applied questionnaires require only verbal and listening skills. Furthermore, the instrument was administered to patients with reduced mobility, blind people and those with low vision, thus promoting an inclusive perspective consistent with the principles of dignity and equity. This strategy is aligned with the World Health Organization’s recommendations in the framework of integrated, person-centered health services, which emphasize the importance of sensitive, respectful care oriented to individual needs and preferences.^26^

The validation of the new instrument included the active participation of patients from the piloting stage. This was carried out directly by the research team, without the intermediation of external staff, which facilitated direct observation of the response process, the identification of comprehension difficulties and the timely conduct of cognitive interviews. This methodology is consistent with that proposed by Willis, who highlights the importance of contextualized observation for the detection of flaws in the design of instruments.^14^ From a psychometric perspective, it is relevant to highlight two aspects: firstly, homogeneity was observed in the socio-demographic characteristics of the participants in the different phases of the study; secondly, in Stage 1, the format of four response options generated an asymmetrical distribution, with concentrations at the upper extremes ("almost always" and "always"). This lack of variability is known to introduce distortions in the calculation of traditional correlations (Pearson and Spearman), which, with results concentrated in two responses, results in coefficients close to 1.0. The specialized literature^18^ recommends the use of five or more response categories to apply polychoric correlations. In this context, a seven-point scale was chosen, consistent with the rating system widely used in Chilean education, which facilitated patient familiarity with the response format. As expected, the polychoric correlations showed more stable behavior, which favored the reliability of the exploratory factor analysis (EFA) and confirmatory factor analysis (CFA).

During this adjustment process, evidence emerged to support the existence of a single latent factor for assessing humanized care. This finding differs from previous studies that identified 32 four-choice Likert-scale items as being explained by three factors.^9^^,^^27^ However, the research team opted for a conservative approach, moving to a third stage of validation with a final version of 15 items and seven response options, applied to a large sample of 1,134 patients. This number gives the instrument a robust basis for use, ensuring efficiency in its application, accessibility for patients with physical or educational limitations, and congruence with the principles of humanized care, particularly in the inpatient setting. It should be noted that although item 8 ("They call you by your name") presented a factor load less than 0.5, it was decided to retain it due to its symbolic and ethical relevance for nursing. This item represents a fundamental component of dignified treatment in health care, supported by current Chilean legislation in Law No. 20,584 on patients' rights and duties,^28^ and its inclusion reaffirms the instrument's commitment to the values of respect and humanization that underpin nursing practice.

A significant reflection made by the research team after the completion of this validation process is the relevance of carrying out the cognitive interviews with the patients and having participated in this process by verbally gathering important comments and points of view, which allowed for the cohesion of an instrument that was more relevant to a wide range of participants. This new instrument proposal, designed to be applied during hospitalization, allows the patient to be surveyed in the natural environment in which he/she experiences the nursing care provided by the staff, which allows for reliable and quality responses. The team of surveyors, made up of nursing professionals with postgraduate training, progressively systematized the data collection procedure through regular meetings. The experience accumulated during this process will be reflected in the elaboration of a manual for the application of the instrument, so that it can be used in a standardized way in any hospital in Chile, provided in similar conditions to those under which the present study was developed.

Conclusion: While the primary objective was to validate a new 32-item version of the PCHE, the validation process ultimately led to the development of an abbreviated 15-item version, which was demonstrated to be valid and reliable for application among Chilean hospitalized patients.

Limitations of the study: Although the sample was not random, it is considered that patients become hospitalized in a random manner.

Grant and subsidy information: This study was funded by Internal Research Funds, 2024, from the Universidad Viña del Mar. FIIUVM-LPI-2402.

Appendix. Scale for Perception of Humanised Care Behaviours in Spanish, abbreviated to 15 items. 

1- Lo (a) hacen sentir como una persona.

2- Lo (la) tratan con amabilidad.

3- Lo (la) miran a los ojos cuando le hablan.

4- Le generan confianza cuando lo(la) cuidan.

5- Le explican previamente los procedimientos que realizarán.

6- Responden con seguridad y claridad a sus preguntas.

7- Le dan explicaciones sobre su atención de salud cuando usted lo pide.

8- Lo (la) llaman por su nombre.

9- Atienden oportunamente sus necesidades básicas (higiene, alimentación, evacuación urinaria e intestinal).

10- Le permiten expresar sus sentimientos sobre la enfermedad y tratamiento.

11- Responden oportunamente a su llamado.

12- Lo (la) escuchan atentamente.

13- Le preguntan y se preocupan por su estado de ánimo.

14- Lo (la) ayudan a manejar su dolor físico.

15- Le respetan su intimidad física.

## References

[1] Romero E, Contreras IM, Moncada A (2016). Relación entre cuidado humanizado por enfermería con la hospitalización de pacientes. Hacia la Promoción de la Salud.

[2] Jimenez LA, Gamboa R, Márquez M (2019). Deshumanización en la atención de la salud ¿son las Tic´s el problema o la solución?. Mundo Fesc.

[3] Corbani NM de S, Brêtas ACP, Matheus MCC (2009). Humanização do cuidado de enfermagem: o que é isso?. Revista Brasileira de Enfermagem.

[4] Paterson JG, Zderad LT (2008). Humanistic nursing.

[5] Watson J (2012). Human caring science: a theory of nursing.

[6] Blanco-Nistal M, Tortajada-Soler M, Rodriguez-Puente Z, Puente-Martínez M, Méndez-Martínez C, Fernández-Fernández J (2021). Percepción de los pacientes sobre los cuidados de enfermería en el contexto de la crisis del COVID-19. Enfermería Global.

[7] Ghanbari-Afra L, Adib-Hajbaghery M, Dianati M (2022). Human Caring: A Concept Analysis. Journal of Caring Sciences.

[8] Rivera-Álvarez L, Triana Á (2023). Proceso de construcción y validación del instrumento Percepción de Comportamientos de Cuidado Humanizado de Enfermería (PCHE-III). Index de Enfermería.

[9] Melita Rodríguez A, Jara Concha P, Pereira DI, Luengo Machuca L (2018). Adaptación transcultural y validación de un cuestionario de cuidado humanizado en enfermería para una muestra de población Chilena. Revista Cuidarte.

[10] Campo-Arias A, Oviedo HC (2008). Propiedades Psicométricas de una Escala: la Consistencia Interna. Revista de Salud Pública.

[11] Comrey A L, Lee HB A First Course in Factor Analysis.

[12] Kline RB (2016). Principles and practice of structural equation modeling.

[13] Sánchez R (2021). El tema de validez de contenido en la educación y la propuesta de Hernández-Nieto. Latin American Journal of Physical Education.

[14] Willis GB (2005). Cognitive interviewing: A tool for improving questionnaire design.

[15] Tourangeau R, Rips LJ, Rasinski K (2000). The psychology of survey response.

[16] Collins D (2003). Pretesting survey instruments: an overview of cognitive methods. Quality of Life Research.

[17] Geerts M, Hoeijmakers JGJ Essers M (2024). Development, validation and feasibility of a Patient Satisfaction Questionnaire for evaluating the quality performance of a diagnostic small fibre neuropathy service: A qualitative study. Health Expectations.

[18] Kiwanuka F, Kopra J, Sak-Dankosky N, Nanyonga RC, Kvist T (2022). Polychoric Correlation with Ordinal Data in Nursing Research. Nursing Research.

[19] Holgado-Tello FP, Chacón-Moscoso S, Barbero-García I (2010). Polychoric versus Pearson correlations in exploratory and confirmatory factor analysis of ordinal variables. Quality & Quantity.

[20] Mardia K, Kent J, Taylor C (2024). Multivariate Analysis.

[21] Finch WH, French BF (2015). Latent Variable Modeling with R.

[22] Kost RG, de Rosa JC (2018). Impact of survey length and compensation on validity, reliability, and sample characteristics for Ultrashort-, Short-, and Long-Research Participant Perception Surveys. Journal of Clinical and Translational Science.

[23] Vaus DD (2013). Surveys in Social Research (Social Research Today).

[24] Recart A (2017). Cirugía mayor ambulatoria. Una nueva forma de entender la medicina quirúrgica. Revista Médica Clínica Las Condes.

[25] Sharma H (2022). How short or long should be a questionnaire for any research? Researchers dilemma in deciding the appropriate questionnaire length. Saudi Journal of Anesthesiology.

[26] 26. World Health Organization (WHO). Integrated people-centred care [Internet]. Available from: https://www.who.int/health-topics/integrated-people-centered-care#tab=tab_1

[27] Monje P, Miranda P, Oyarzún J, Seguel F, Flores E (2018). Percepción de cuidado humanizado de enfermería desde la perspectiva de usuarios hospitalizados. Ciencia y Enfermería.

[28] Ministerio de salud de Chile, Subsecretaria de Salud Pública (2012). Ley Nº 20.584: Regula los derechos y deberes que tienen las personas en relación con acciones vinculadas a su atención en salud.

